# Outcomes of Different Reperfusion Strategies of Multivessel Disease Undergoing Newer-Generation Drug-Eluting Stent Implantation in Patients with Non-ST-Elevation Myocardial Infarction and Chronic Kidney Disease

**DOI:** 10.3390/jcm10204629

**Published:** 2021-10-09

**Authors:** Yong Hoon Kim, Ae-Young Her, Myung Ho Jeong, Byeong-Keuk Kim, Sung-Jin Hong, Seung-Jun Lee, Chul-Min Ahn, Jung-Sun Kim, Young-Guk Ko, Donghoon Choi, Myeong-Ki Hong, Yangsoo Jang

**Affiliations:** 1 Division of Cardiology, Department of Internal Medicine, Kangwon National University School of Medicine, Chuncheon 24289, Korea; hermartha1@gmail.com; 2 Department of Cardiology, Cardiovascular Center, Chonnam National University Hospital, Gwangju 61469, Korea; myungho@chollian.net; 3 Division of Cardiology, Severance Cardiovascular Hospital, Yonsei University College of Medicine, Seoul 03722, Korea; kimbk@yuhs.ac (B.-K.K.); HONGS@yuhs.ac (S.-J.H.); SJUNLEE@yuhs.ac (S.-J.L.); DRCELLO@yuhs.ac (C.-M.A.); kjs1218@yuhs.ac (J.-S.K.); ygko@yuhs.ac (Y.-G.K.); cdhlyj@yuhs.ac (D.C.); mkhong61@yuhs.ac (M.-K.H.); 4 Department of Cardiology, CHA Bundang Medical Center, CHA University School of Medicine, Seongnam 13496, Korea; jangys1212@cha.ac.kr

**Keywords:** angioplasty, drug-eluting stents, non-ST-elevation myocardial infarction, multivessel disease

## Abstract

Because available data are limited, we compared the 2-year clinical outcomes among different reperfusion strategies (culprit-only percutaneous coronary intervention (C-PCI), multivessel PCI (M-PCI), complete revascularization (CR) and incomplete revascularization (IR)) of multivessel disease (MVD) undergoing newer-generation drug-eluting stent implantation in patients with non-ST-elevation myocardial infarction (NSTEMI) and chronic kidney disease (CKD). In this nonrandomized, multicenter, retrospective cohort study, a total of 1042 patients (C-PCI, *n* = 470; M-PCI, *n* = 572; CR, *n* = 432; IR, *n* = 140) were recruited from the Korea Acute Myocardial Infarction Registry (KAMIR) and evaluated. The primary outcome was the occurrence of major adverse cardiac events, defined as all-cause death, recurrent myocardial infarction and any repeat coronary revascularization. The secondary outcome was probable or definite stent thrombosis. During the 2-year follow-up period, the cumulative incidences of the primary (C-PCI vs. M-PCI, adjusted hazard ratio (aHR), 1.020; *p* = 0.924; CR vs. IR, aHR, 1.012; *p* = 0.967; C-PCI vs. CR, aHR, 1.042; *p* = 0.863; or C-PCI vs. IR, aHR, 1.060; *p* = 0.844) and secondary outcomes were statistically insignificant in the four comparison groups. In the contemporary newer-generation DES era, C-PCI may be a better reperfusion option for patients with NSTEMI with MVD and CKD rather than M-PCI, including CR and IR, with regard to the procedure time and the risk of contrast-induced nephropathy. However, further well-designed, large-scale randomized studies are warranted to confirm these results.

## 1. Introduction

The extent of coronary artery disease (CAD) is a marker of diffuse atherosclerosis and plaque burden and multivessel disease (MVD) is associated with worse outcomes in patients with infarction (AMI) [1]. The incidence of MVD in patients with non-ST-segment elevation myocardial infarction (NSTEMI) is more than 50% [2,3]. Even though percutaneous coronary intervention (PCI) for an infarct-related artery (IRA) is a well-established standard treatment [4,5], the treatment strategies for a non-IRA in the NSTEMI milieu are still debatable [6,7,8,9]. Revascularization of the non-IRA may reduce the incidence of recurrent ischemia, improve left ventricular function, reduce arrhythmias and potentially improve hemodynamics [10]. In contrast, procedural complexity might lead to overexposure to radiation and an increased risk of developing contrast-induced nephropathy and further ischemia [11,12,13] in patients with AMI and MVD. Approximately 25–30% of patients with NSTEMI have moderately reduced renal function [14]. A drop of 10 mL/min/1.73 m^2^ in the glomerular filtration rate (GFR) leads to a 5% to 6% incremental increase in cardiovascular mortality rates [15]. Thus, patients with chronic kidney disease (CKD) and NSTEMI have worse prognosis than those with normal renal function [16]. Unfortunately, individuals with CKD are often excluded from or underrepresented in randomized trials and are less likely to receive guideline-recommended medical and revascularization therapy [17]. Yet, data on PCI patients with NSTEMI with MVD and CKD are limited. Additionally, according to a recent meta-analysis, the use of second-generation drug-eluting stent (2G-DES) resulted in an 18% reduction in all-cause death and a 27% reduction in target lesion revascularization/target vessel revascularization (TLR/TVR) compared to the use of first-generation DES (1G-DES) in patients with CKD [18]. Hence, after confining the study population who received newer-generation DES to reflect current real-world practice, we compared the 2-year clinical outcomes among different reperfusion strategies (culprit-only PCI (C-PCI), multivessel PCI (M-PCI), complete revascularization (CR) and incomplete revascularization (IR)) of MVD in patients with NSTEMI and CKD.

## 2. Methods

### 2.1. Study Population

**Figure 1:**
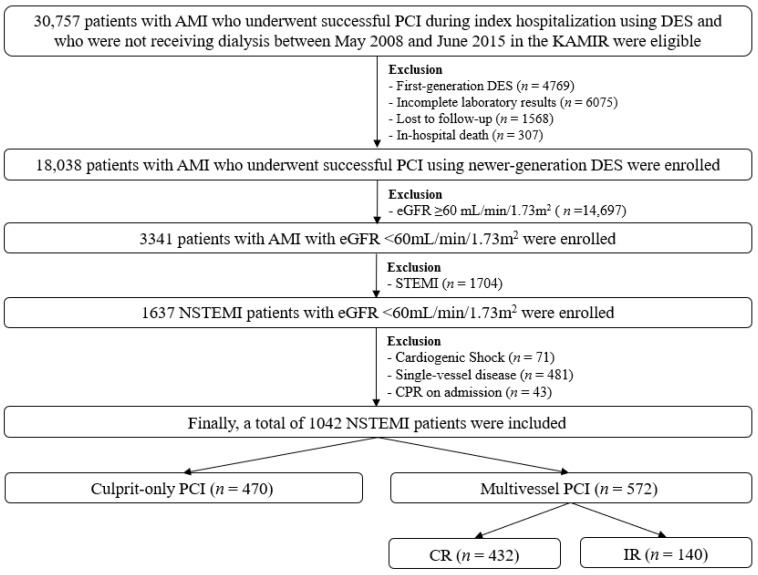
Flowchart. AMI, acute myocardial infarction; PCI, percutaneous coronary intervention; DES, drug-eluting stent; KAMIR, Korea AMI Registry; eGFR, estimated glomerular filtration rate; STEMI, ST-segment-elevation myocardial infarction; NSTEMI, non-STEMI; CPR, cardiopulmonary resuscitation; CR, complete revascularization; IR, incomplete revascularization.

In this nonrandomized, multicenter, retrospective cohort study, a total of 30,757 patients with AMI who underwent successful PCI during index hospitalization using DES and who were not receiving continuous renal replacement therapy, including hemodialysis or peritoneal dialysis, between May 2008 and June 2015 were recruited from the Korea AMI Registry (KAMIR) [19]. KAMIR is the first nationwide and multicenter registry that included >50 tertiary-care teaching hospitals in South Korea since November 2005. Detailed information on this registry can be found on the website (http://www.kamir.or.kr (accessed on 6 May 2021). Eligible patients were aged ≥18 years at the time of hospital admission. Patients with the following were also excluded: deployed 1G-DES (*n* = 4769, 15.5%), incomplete laboratory results (*n* = 6075, 19.8%), loss to follow-up (*n* = 1568, 5.1%) and in-hospital death (*n* = 307, 1.0%). A total of 18,038 patients with AMI who underwent successful PCI using newer-generation DES were enrolled. The types of newer-generation DESs used are listed in Table 1. After excluding those with estimated GFR (eGFR) ≥60 mL/min/1.73 m^2^ (*n* = 14,697, 81.5%), 3341 patients (18.5%) with AMI with eGFR <60 mL/min/1.73 m^2^ remained. After excluding those with STEMI (*n* = 1704, 51%), 1637 patients (49%) with NSTEMI remained. Those with cardiogenic shock (*n* = 71, 4.3%), single-vessel disease (*n* = 481, 29.4%), and cardiopulmonary resuscitation (CPR) on admission (*n* = 43, 2.6%) were also excluded. Finally, 1042 patients with NSTEMI were included in the study. Patients were assigned to the C-PCI (*n* = 470, 45.1%) and M-PCI (*n* = 572, 54.9%) groups. In the case of M-PCI, 432 (75.5%) patients received CR and 140 (24.5%) patients received IR (Figure 1). All data were collected using a web-based case report form at each participating center. The study was conducted in accordance with the ethical guidelines of the 2004 Declaration of Helsinki and was approved by the ethics committee of each participating center and the Chonnam National University Hospital Institutional Review Board ethics committee (CNUH-2011-172). All 1042 patients included in the study provided written informed consent prior to enrollment. They also completed a 2-year clinical follow-up through face-to-face interviews, phone calls or chart reviews. The processes of event adjudication have been described in a previous publication by KAMIR investigators [20].

### 2.2. Percutaneous Coronary Intervention and Medical Treatment

Following general guidelines [21], coronary angiography and PCI were performed via a transfemoral or transradial approach. Aspirin (200–300 mg) and clopidogrel (300–600 mg) when available, or ticagrelor (180 mg) or prasugrel (60 mg), were prescribed as loading doses to the individuals before PCI. After PCI, dual antiplatelet therapy (DAPT; a combination of aspirin (100 mg/day) with clopidogrel (75 mg/day) or ticagrelor (90 mg twice a day) or prasugrel (5–10 mg/day)) was recommended at least 12 months. Based on previous reports [22,23], triple antiplatelet therapy (TAPT; 100 mg of cilostazol was administered twice a day in addition, to DAPT) was administered at the discretion of the individual operator. Moreover, the access site, revascularization strategy and selection of DES were left to the discretion of the individual operators.

### 2.3. Study Definitions and Clinical Outcomes

Glomerular function was calculated using the Chronic Kidney Disease Epidemiology Collaboration equation for eGFR [24]. In this study, CKD was defined as eGFR <60 mL/min/1.73 m^2^ [25,26]. If the patients showed the absence of persistent ST-segment elevation with increased cardiac biomarkers and if the clinical context was appropriate, these patients were considered to have NSTEMI [4,27]. MVD was defined as at least two major vessels (≥2 mm diameter) with >70% stenosis of the vessel diameter [28]. Successful PCI was defined as residual stenosis <30% and thrombolysis in myocardial infarction grade III flow in the IRA after the procedure. The culprit vessel was evaluated by coronary angiographic findings, 12-lead electrocardiogram, two-dimensional echocardiogram and noninvasive stress test [29]. The M-PCI group comprised patients who underwent PCI of the non-IRA during index PCI of the IRA or who underwent staged PCI for the non-IRA within the index hospitalization. Hence, patients with NSTEMI and MVD who underwent staged PCI after discharge were excluded from this study (Figure 1). CR was defined as open IRA followed by dilatation of all other significantly narrowed arteries during the primary procedure or index hospitalization. IR was defined as successfully opened IRA followed by dilatation of only the significantly narrowed artery in ≥1 non-IRA vessel during the primary procedure or index hospitalization [30]. The Global Registry of Acute Coronary Events (GRACE) risk score [31] was calculated for all patients. The primary clinical outcome of this study was the occurrence of major adverse cardiac events (MACE), defined as all-cause death, recurrent myocardial infarction (re-MI), or any coronary repeat revascularization, including TLR, TVR and non-TVR. The secondary clinical outcome was definite or probable stent thrombosis (ST) during the 2-year follow-up period. All-cause death was considered cardiac death (CD) unless an undisputed noncardiac cause was present [32]. Any repeat revascularization was composed of TLR, TVR and non-TVR. The definitions of re-MI, TLR, TVR and non-TVR have been previously published [33,34]. The cumulative incidence of ST was defined according to the Academic Research Consortium [35]. However, the incidence of ST in this study, was low; hence, the total number of ST events was described instead of a separate cumulative incidence according to their time interval (acute, subacute, late and very late).

### 2.4. Statistical Analyses

For continuous variables, intergroup differences were evaluated using the unpaired *t*-test and data were expressed as mean ± standard deviation. For categorical variables, intergroup differences were analyzed using the χ^2^ test or, if not applicable, Fisher’s exact test and data were expressed as counts and percentages. Various clinical outcomes were estimated using the Kaplan–Meier method and intergroup differences were compared using the log-rank test. Significant confounding covariates (*p* < 0.05) were included in the multivariate Cox regression analysis. The variables included in the comparison between C-PCI and M-PCI were as follows: age; male sex; left ventricular ejection fraction (LVEF) <40%; blood levels of peak creatine kinase-myocardial band (CK-MB), peak troponin-I, N-terminal pro-brain natriuretic peptide (NT-proBNP) and triglyceride; discharge medications (cilostazol and lipid-lowering agent); IRA (left main coronary artery (LM)); treated vessels (LM, left anterior descending (LAD) artery, left circumflex artery (LCx) and right coronary artery (RCA)); use of intravascular ultrasound (IVUS); time from admission to PCI; number of deployed stents; and GRACE risk score. The variables included in the comparison between CR and IR, between C-PCI and CR and between C-PCI and IR are shown in Table 2. For all analyses, two-sided values of *p* < 0.05 were considered statistically significant. All statistical analyses were performed using the Statistical Package for the Social Sciences version 20 (IBM, Armonk, NY, USA).

## 3. Results

### 3.1. Baseline Characteristics

The baseline clinical, laboratory and procedural characteristics of the study population are summarized in Table 1 and Table S1. In the comparison between C-PCI and M-PCI, the number of male patients and the mean value of peak CK-MB were higher in the C-PCI group and the mean time interval from admission to PCI, the prescription rate of lipid-lowering agent as a discharge medication, IRA (LM) and use of IVUS were significantly higher in the M-PCI group. In the comparison between CR and IR, the mean value of LVEF, the number of 2-vessel disease and American College of Cardiology/American Heart Association (ACC/AHA) type B2 lesion were higher in the CR group. In contrast, the number of patients with hypertension, diabetes mellitus (DM), previous history of PCI and coronary artery bypass graft (CABG), IRA (LM), ≥3-vessel disease and ACC/AHA type B2 lesion were higher in the IR group. In the comparison between C-PCI and CR, the number of male patients, those with a previous history of PCI and the mean value of peak CK-MB were higher in the C-PCI group. However, the mean time from admission to PCI, number of all treated vessels (LM, LAD, LCx and RCA), use of IVUS and mean diameter of deployed stent were higher in the CR group. In the comparison between C-PCI and IR, the mean value of LVEF and the number of ACC/AHA type B2 lesion were higher in the C-PCI group. The number of DM, lipid-lowering agents as a discharge medication, IRA (LM), all treated vessels, ≥3-vessel disease, use of IVUS and mean number of deployed stents were higher in the IR group. The mean value of the GRACE risk score and the number of patients with GRACE risk score >140 were similar between the C-PCI and M-PCI groups, between the CR and IR groups, between the C-PCI and CR groups and between the C-PCI and IR groups (Table 1 and Table S1).

### 3.2. Clinical Outcomes

**Figure 2:**
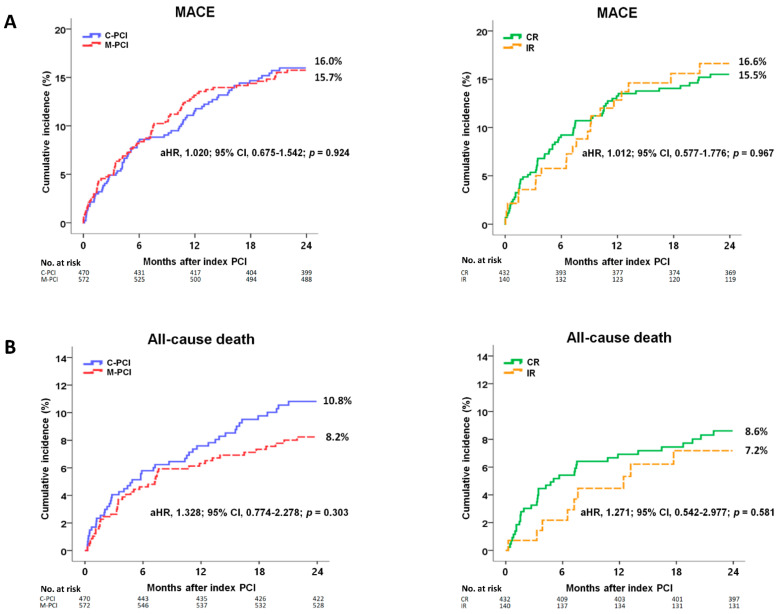

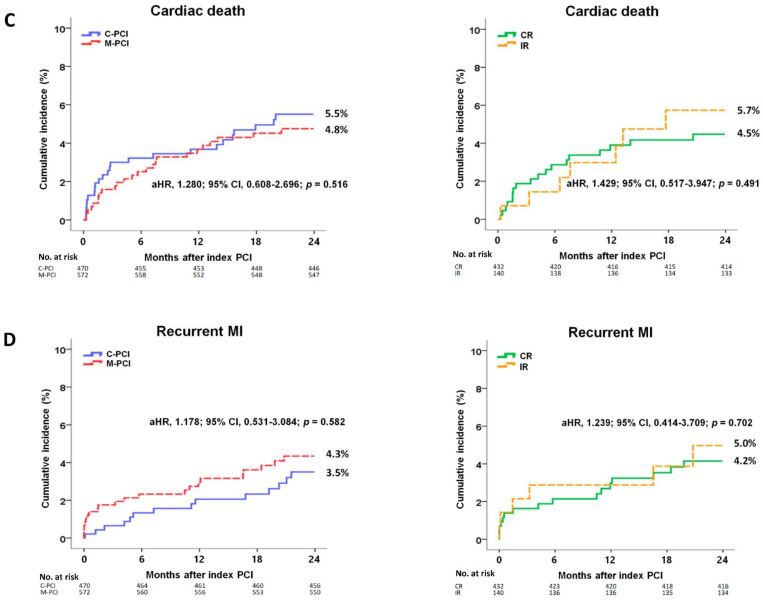

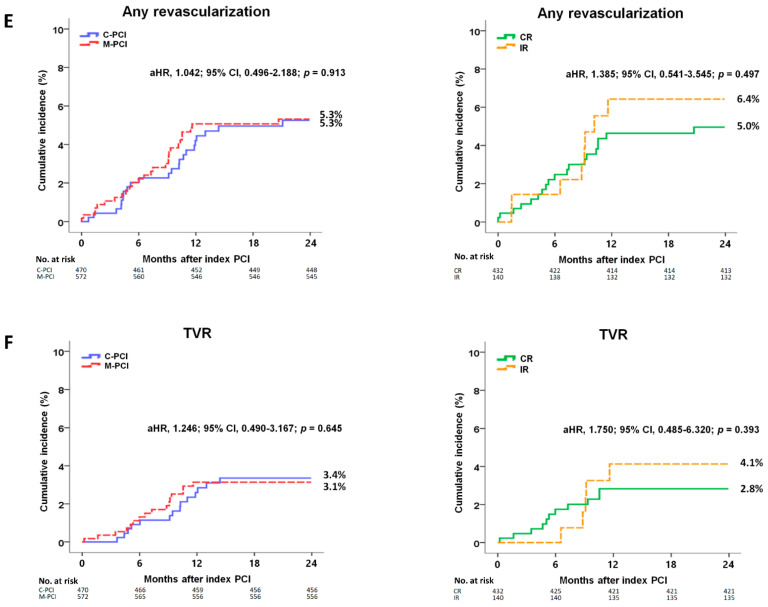

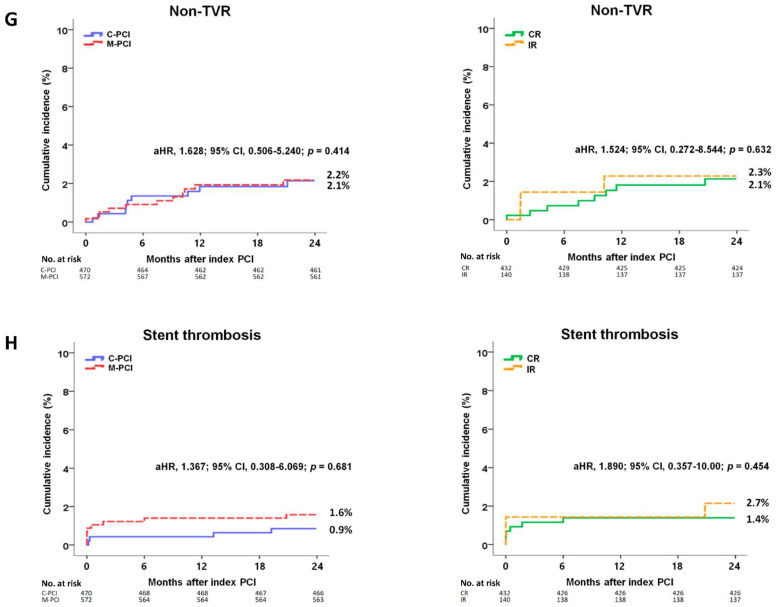
Kaplan-Meier analyses for the MACE (**A**), all-cause death (**B**), cardiac death (**C**), recurrent MI (**D**), any repeat revascularization (**E**), TVR (**F**), non-TVR (**G**) and ST (**H**) between the C-PCI group and the M-PCI group and the CR group and the IR group at 2 years. aHR, adjusted hazard ratio; CI, confidence interval; MACE, major adverse cardiac events; MI, myocardial infarction; TVR, target vessel revascularization; C-PCI, culprit-only PCI; M-PCI, multivessel PCI; CR, complete revascularization; IR, incomplete revascularization.

The cumulative incidences of major clinical outcomes at 2 years are listed in Table 2, Figure 2 and Figure S1. In the comparison between C-PCI and M-PCI, after adjustment, the cumulative incidences of MACE (Figure 2A), all-cause death (Figure 2B), CD (Figure 2C), re-MI (Figure 2D), any repeat revascularization (Figure 2E), TVR (Figure 2F), non-TVR (Figure 2G) and ST (Figure 2H) were similar between the C-PCI and M-PCI groups. In the comparison between CR and IR, after adjustment, the cumulative incidences of all major clinical outcomes were similar between the CR and IR groups. Similarly, the primary and secondary clinical outcomes were similar between the C-PCI and CR groups and between the C-PCI and IR groups (Table 2 and Figure S1). Table 3 shows the independent predictors of MACE at 2 years. Reduced LVEF (<40%), peak troponin-I and NT-proBNP levels were significant independent predictors of MACE.

## 4. Discussion

The main findings of this study are as follows: (1) the cumulative incidence rates of MACE, all-cause death, CD, re-MI, any repeat revascularization, TVR, non-TVR and ST were similar between the C-PCI and M-PCI groups, between the CR and IR groups, between the C-PCI and CR groups, and between the C-PCI and IR groups and (2) reduced LVEF (<40%), CPR on admission and peak troponin-I and NT-proBNP levels were significant independent predictors of MACE.

Patients with NSTEMI tend to have MVD and more complex disease than patients with STEMI [36]. Although the current guidelines recommend an early invasive strategy in patients with high-risk NSTEMI [4,5], the optimal treatment strategy for NSTEMI with MVD is still debatable. Recently, Rathod et al. [9] showed that single-stage CR appears to be superior to C-PCI in terms of long-term mortality (22.5% vs. 25.9, *p* = 0.0005) during a median of 4.1-year follow-up period in their 21,857 NSTEMI patients with MVD. This study has a large sample size, provides adequate power and is very valuable because it shows the mortality reduction capability of single-stage CR. However, about 24% of the enrolled patients received bare-metal stents (BMS) and the number of patients who received newer-generation DES is unclear. In the era of newer-generation DES, BMS is rarely used and 1G-DES is nearly replaced with a thinner and more biocompatible or biodegradable polymer-coated newer-generation DES with better clinical outcomes [30]. Furthermore, as mentioned, 2G-DES was beneficial in reducing mortality and TLR/TVR in patients with CKD [18]. The current guidelines also recommend newer-generation DES over BMS during PCI in patients with NSTE-acute coronary syndrome (ACS) and CKD [5]. Thus, their findings have some limitations in reflecting the current real-world practice. In the Impact of Different Treatment in Multivessel Non-ST-Elevation Myocardial Infarction (NSTEMI) Patients: One Stage Versus Multistaged PCI (SMILE) randomized trial [37], the 1-year rate of major adverse cardiovascular and cerebrovascular events was lower in the one-stage coronary revascularization group than that in the multistage PCI group (13.63% vs. 23.19%, *p* = 0.004). In their study [37], the number of patients who received BMS or plain old balloon angioplasty was approximately 18%. More recently, Liu et al. [6] demonstrated that immediate M-PCI was associated with worse long-term outcomes than stage M-PCI during index admission (log-rank *p* < 0.001). However, their study included about 40% of STEMI patients and the deployed stents were not confined to newer-generation DES. Similarly, other previous studies [7,8] also included patients who received BMS or 1G-DES. Additionally, studies concerning long-term outcomes according to different reperfusion strategies in patients with NSTEMI with MVD and CKD after PCI using newer-generation DES are limited.

Although CKD patients have frequent risk factors and comorbidities, many large-scale trials have excluded patients with CKD [17]. Hence, the long-term effects of revascularization therapy in these patients are not fully understood. A previous report [38] suggested that early revascularization could reduce the risk of 1-year mortality compared to initial medical therapy (odds ratio [OR], 0.46; *p* = 0.008) in 23,234 ACS patients. The most recent meta-analysis [39] demonstrated that PCI cannot improve short- (≤1 month, OR, 0.65; *p* = 0.079) and medium-term (1 month to 1 year, OR, 0.70; *p* = 0.157) all-cause death compared with medical treatment in patients with AMI. The American guideline [4] recommends that an invasive strategy is reasonable in patients with NSTE-ACS with mild (stage 2) and moderate (stage 3) CKD (class IIa, level of evidence B). According to the European guideline [5], PCI should be considered for CABG in patients with NSTE-ACS and CKD with MVD whose surgical risk profile is high or the life expectancy is <1 year (class IIa, level of evidence B). Current evidence [40] does not recommend routine immediate M-PCI in AMI patients with cardiogenic shock. Therefore, the remaining issue concerns AMI patients with MVD without cardiogenic shock, which is considered an ongoing issue for interventional cardiologists [39]. As shown in Figure 1, patients with cardiogenic shock were excluded in our study. Patients with NSTEMI and cardiogenic shock have worse clinical outcomes than those with STEMI and cardiogenic shock [41] and PCI of the non-IRA may aggravate hemodynamic instability and jeopardize the viable myocardium in the milieu of AMI [6].

In our study, regarding baseline characteristics (Table 1), in the M-PCI group, the mean value of triglycerides, the number of LM as an IRA, the number of treated vessels (LM, LAD, LCx and RCA) and the mean time from admission to PCI were higher than that in the C-PCI group. The number of patients with hypertension, DM, previous PCI, previous CABG, LM as an IRA and ≥3-vessel disease was higher in the IR group than that in the CR group. Moreover, the mean value of LVEF was also lower in the IR group than that in the CR group (46.1% ± 12.5% vs. 49.7% ± 12.7%, *p* = 0.010). In the C-PCI group, the number of patients with previous PCI and the mean value of peak CK-MB were higher than that in the CR group. However, the number of patients with DM, LM as IRA and ACC/AHA type B2 lesions was higher in the IR group than that in the C-PCI group. Additionally, the mean value of LVEF was lower in the IR group than that in the C-PCI group (46.1% ± 12.5% vs. 48.3% ± 12.6%, *p* = 0.037, Table S1). Although baseline characteristics were significantly different between the four groups (C-PCI vs. M-PCI, CR vs. IR, C-PCI vs. CR and C-PCI vs. IR), the 2-year major clinical outcomes were not significantly different between these groups (Table 2). Although we could not precisely determine the etiologic factors for these results, one possible explanation may be related to the similar distribution of significant independent predictors for MACE (Table 3, reduced LVEF <40%, peak troponin-I and NT-proBNP levels) in these comparison groups. Recently, Kim et al. [42] reported that the cumulative incidences of major clinical outcomes were similar in the three comparison groups (C-PCI vs. M-PCI, CR vs. IR, or C-PCI vs. CR) except for non-TVR in 4588 patients with NSTEMI and MVD after newer-generation DES implantation. They mentioned that the higher incidence rate of non-TVR in the C-PCI group may be related to the initial selection of treatment strategies, that is, either C-PCI or M-PCI, during the index PCI. As this selection was based on the physician’s preference, in the C-PCI group, regardless of whether the lesions were considered significantly invasive during the initial procedure, these lesions were not treated. As a result, the PCIs were possibly included as non-TVR in the C-PCI group. However, in their study [42], patients with cardiogenic shock were included and the enrolled patients were not confined to those with CKD. Because there are very limited studies that can be used to directly compare the results of our study, determining the value of this study in comparison to that of other studies and speculating the main cause of the results of this study compared to those of other studies are challenging.

Regarding patients with STEMI, Mehta et al. [43] demonstrated that CR was superior to C-PCI in patients with STEMI and MVD in reducing cardiovascular death or MI, as well as the risk of cardiovascular death, MI, or ischemia-driven revascularization in their randomized trial. However, in the more recent review [44], a strategy of staged PCI of obstructive non-culprit lesions should be considered the gold standard for the treatment of patient with STEMI and MVD. However, what is the optimal timing of staged PCI is not completely defined and the assessment of intermediate non-culprit lesions is still a major problem [44]. Moreover, they [44] also mentioned that there are no studies demonstrating that preventive PCI of vulnerable plaques or more intensive pharmacological treatment is associated with an improved clinical outcome.

Patients with CKD have a high prevalence of DM and an increased chance of having 3-vessel CAD, LM disease and coronary calcification [45]. As the severity of CKD progresses, the severity and extent of CAD also increases [46]. Therefore, patients with CKD undergoing PCI need to carefully consider diverse clinical options to minimize the risk of contrast-induced nephropathy and optimize clinical outcomes [47]. In real-world practice, despite the limitation in available data, CKD patients presenting with NSTEMI with MVD received the same approach as those with normal renal function [48]. With respect to these limitations of current practice in patients with NSTEMI with MVD and CKD, we believe that our results could be helpful to interventional cardiologists in terms of providing current real-world information regarding clinical outcomes among different multivessel reperfusion strategies in patients with NSTEMI and CKD. Furthermore, although the study population was insufficient to show meaningful results, more than 50 high-volume tertiary-care teaching hospitals in South Korea participated in this study.

This study had other limitations. First, because of the retrospective nature of this cohort study, there may have been some underreporting and/or missing data and selection bias. Second, CKD is strongly associated with an increased risk of bleeding in patients undergoing PCI [49]. However, because the value of this variable was incomplete due to missing values, we could not include this as a meaningful variable in our study. Therefore, this was a major limitation of this study. Third, the estimation of renal function was based on a single measurement of eGFR at the time of presentation to the hospital. Therefore, there is a possibility that eGFR may have worsened during the follow-up period. Unfortunately, we could not provide follow-up eGFR values because of the limitations of the registry data. Fourth, the variables that were not included in the data registry might have affected the study outcome. Fifth, although the time interval from symptom onset to PCI is an important determinant of major clinical outcomes, this variable included many missing values in the registry data. Therefore, we could not include this variable in the present study, which may have resulted in bias. Sixth, the 2-year follow-up period in this study was relatively short for estimating long-term clinical outcomes. Seventh, our study was focused on patients with CKD, so it is intuitive to have a primary or secondary outcome including for example need for renal replacement therapy during hospitalization, or occurrence of contrast-induced nephropathy. However, because these variables were not mandatory variables, we could not include these variables as the major outcomes in this study. This point was other important limitation of our study. Eighth, because limitations of medical insurance system in Korea, the use of fractional flow reserve/instant wave-free ratio was very restricted in this study (Table 1). Thus, in this study, the patients with intermediate stenotic lesions were not fully evaluated. Finally, this study enrolled patients who underwent PCI between May 2008 and June 2015 and this broad timeframe could have affected the clinical outcomes.

## 5. Conclusions

In the contemporary newer-generation DES era, our results suggest that C-PCI may be a better option for patients with NSTEMI with MVD and CKD rather than M-PCI, including CR and IR, with regard to procedure time and the risk of contrast-induced nephropathy. However, further well-designed, large-scale randomized studies are warranted to confirm these results.

## Figures and Tables

**Table 1:** Baseline clinical, laboratory, angiographic and procedural characteristics.

Variables	Culprit-Only PCI (*n* = 470)	Multivessel PCI (*n* = 572)	*p* Value	CR (*n* = 432)	IR (*n* = 140)	*p* Value
Age (years)	71.7 ± 9.7	71.3 ± 9.1	0.431	71.3 ± 9.2	71.2 ± 9.0	0.876
≥65 years, *n* (%)	364 (77.4)	434 (75.9)	0.551	328 (75.9)	106 (75.7)	0.959
Male, *n* (%)	278 (59.1)	298 (52.1)	0.023	217 (50.2)	81 (57.9)	0.117
LVEF (%)	48.3 ± 12.6	49.1 ± 12.8	0.283	49.9 ± 12.7	46.1 ± 12.5	0.010
<40%, *n* (%)	116 (24.7)	131 (22.9)	0.502	93 (21.5)	38 (27.1)	0.169
BMI (kg/m^2^)	23.6 ± 3.3	23.8 ± 3.3	0.354	23.9 ± 3.3	23.54 ±3.3	0.081
SBP (mmHg)	133.9 ± 30.1	134.73 ± 29.7	0.664	134.5 ± 29.3	135.3 ± 31.0	0.774
DBP (mmHg)	78.3 ± 16.6	78.1 ± 15.5	0.884	78.1 ± 15.4	78.3 ± 15.6	0.864
Killip class III, *n* (%)	83 (17.7)	101 (17.7)	0.999	75 (17.4)	26 (18.6)	0.744
Hypertension, *n* (%)	363 (77.2)	439 (76.7)	0.853	323 (74.8)	116 (82.9)	0.049
Diabetes mellitus, *n* (%)	247 (52.6)	325 (56.8)	0.169	234 (54.2)	91 (65.0)	0.025
Dyslipidemia, *n* (%)	294 (62.6)	378 (66.1)	0.236	294 (68.1)	84 (60.0)	0.080
Previous MI, *n* (%)	37 (7.9)	48 (8.4)	0.761	33 (7.6)	15 (10.7)	0.254
Previous PCI, *n* (%)	71 (15.1)	68 (11.9)	0.128	42 (9.7)	26 (18.6)	0.007
Previous CABG, *n* (%)	11 (2.3)	8 (1.4)	0.352	3 (0.7)	5 (3.6)	0.024
Previous HF, *n* (%)	22 (4.7)	25 (4.4)	0.881	18 (4.2)	7 (5.0)	0.640
Previous CVA, *n* (%)	72 (15.3)	73 (12.8)	0.235	54 (12.5)	19 (13.6)	0.771
Current smokers, *n* (%)	98 (20.9)	103 (18.0)	0.247	80 (18.5)	23 (16.4)	0.615
Peak CK-MB (mg/dL)	61.1 ± 96.8	48.8 ± 80.8	0.049	47.6 ± 75.9	52.5 ± 94.5	0.578
Peak troponin-I (ng/mL)	35.6 ± 91.5	26.8 ± 97.2	0.216	24.4 ± 60.0	34.3 ± 88.7	0.491
NT-ProBNP (pg/mL)	7027.3 ± 9781.9	6152.7 ± 9097.5	0.166	5825.1 ± 8862.6	7015.4 ± 8725.4	0.151
Hs-CRP (mg/dL)	9.4 ± 32.9	9.5 ± 40.7	0.962	10.8 ± 45.8	5.5 ± 17.4	0.047
Serum creatinine (mg/L)	2.41 ± 2.45	2.36 ± 2.62	0.729	2.32 ± 2.64	2.45 ± 2.58	0.634
eGFR, mL/min/1.73 m^2^	39.6 ± 16.7	40.2 ± 16.6	0.617	40.8 ± 16.5	38.3 ± 16.8	0.121
Blood glucose (mg/dL)	189.4 ± 100.3	198.5 ± 110.0	0.162	193.6 ± 108.1	213.6 ± 114.7	0.071
Total cholesterol (mg/dL)	169.7 ± 56.8	173.6 ± 46.3	0.224	174.1 ± 45.7	172.1 ± 48.1	0.666
Triglyceride (mg/L)	118.2 ± 71.0	128.6 ± 106.3	0.039	130.9 ± 116.3	121.8 ± 66.0	0.253
HDL cholesterol (mg/L)	42.5 ± 22.2	40.6 ± 10.9	0.088	40.2 ± 10.5	42.0 ± 12.0	0.097
LDL cholesterol (mg/L)	103.8 ± 41.9	107.2 ± 36.1	0.160	109.1 ± 35.4	101.5 ± 38.0	0.038
Discharge medications						
Aspirin, *n* (%)	451 (96.0)	554 (96.9)	0.437	418 (96.8)	136 (97.1)	0.821
Clopidogrel, *n* (%)	435 (92.6)	530 (92.5)	0.862	405 (93.8)	125 (89.3)	0.098
Ticagrelor, *n* (%)	23 (4.9)	31 (5.4)	0.779	19 (4.4)	12 (8.6)	0.083
Prasugrel, *n* (%)	12 (2.6)	11 (1.9)	0.530	8 (1.9)	3 (2.1)	0.735
Cilostazole, *n* (%)	77 (16.4)	138 (24.1)	0.002	121 (28.0)	17 (12.1)	<0.001
Beta-blocker, *n* (%)	368 (78.3)	449 (78.5)	0.938	339 (78.5)	110 (78.6)	0.980
ACEI, *n* (%)	202 (43.0)	233 (40.7)	0.465	180 (41.7)	53 (37.9)	0.489
ARB, *n* (%)	167 (35.5)	214 (37.4)	0.531	158 (36.6)	56 (40.0)	0.467
CCB, n (%)	81 (17.2)	88 (15.4)	0.420	64 (14.8)	24 (17.1)	0.507
Lipid lowering agent, *n* (%)	360 (76.6)	470 (82.2)	0.028	348 (80.6)	122 (87.1)	0.077
Angiographic & procedural characteristics						
IRA						
LM, *n* (%)	17 (3.6)	36 (6.3)	0.048	22 (5.1)	14 (10.0)	0.038
LAD, *n* (%)	190 (40.4)	202 (35.3)	0.104	151 (35.0)	51 (36.4)	0.751
LCx, *n* (%)	105 (22.3)	143 (25.0)	0.316	114 (26.4)	29 (20.7)	0.216
RCA, *n* (%)	158 (33.6)	191 (33.4)	0.939	145 (33.6)	46 (32.9)	0.918
Treated vessel						
LM, *n* (%)	21 (4.5)	56 (9.8)	0.001	38 (8.8)	18 (12.9)	0.160
LAD, *n* (%)	217 (46.2)	420 (73.4)	<0.001	321 (74.3)	99 (70.7)	0.403
LCx, *n* (%)	131 (27.9)	352 (61.5)	<0.001	284 (65.7)	68 (48.6)	<0.001
RCA, *n* (%)	180 (38.3)	317 (55.4)	<0.001	247 (57.2)	70 (50.0)	0.138
Extent of CAD						
2-vessel disease, *n* (%)	229 (48.7)	270 (47.2)	0.663	227 (52.5)	43 (30.7)	<0.001
≥3-vessel disease, *n* (%)	241 (51.3)	302 (52.8)	0.663	205 (47.5)	97 (69.3)	<0.001
ACC/AHA lesion type						
Type B1, *n* (%)	62 (13.2)	78 (13.6)	0.856	54 (12.5)	24 (17.1)	0.164
Type B2, *n* (%)	154 (32.8)	180 (31.5)	0.655	154 (35.6)	26 (18.6)	<0.001
Type C, *n* (%)	224 (47.7)	272 (47.6)	0.973	196 (45.4)	76 (54.3)	0.066
Pre-PCI TIMI flow grade 0/1, *n* (%)	185 (39.4)	228 (39.9)	0.870	179 (41.4)	49 (35.0)	0.197
In-hospital GP IIb/IIIa, *n* (%)	28 (6.0)	25 (4.4)	0.260	17 (3.9)	8 (5.7)	0.371
Drug-eluting stents ^a^						
ZES, *n* (%)	168 (35.7)	203 (35.5)	0.932	164 (38.0)	39 (27.9)	0.033
EES, *n* (%)	248 (52.8)	321 (56.1)	0.279	233 (53.9)	88 (62.9)	0.064
BES, *n* (%)	54 (11.5)	66 (11.5)	0.980	47 (10.9)	19 (13.6)	0.386
Others, *n* (%)	6 (1.3)	7 (1.2)	0.939	6 (1.4)	1 (0.7)	0.528
IVUS, *n* (%)	68 (14.5)	138 (24.1)	<0.001	99 (22.9)	39 (27.9)	0.235
OCT, *n* (%)	1 (0.2)	2 (0.3)	0.682	1 (0.2)	1 (0.7)	0.430
FFR, *n* (%)	3 (0.6)	2 (0.3)	0.502	1 (0.2)	1 (0.7)	0.430
Completeness of multivessel PCI						
CR, *n* (%)	-	432 (75.5)	-	432 (100.0)	-	-
IR, *n* (%)		140 (24.5)	-	-	140 (100.0)	-
PCI for non-IRA	-					
During index PCI, *n* (%)	-	402 (70.3)	-	315 (72.9)	87 (62.1)	0.015
Staged PCI before discharge, *n* (%)	-	170 (29.7)	-	117 (27.1)	53 (37.9)	0.015
Time from admission to PCI (hours)	18.1 ± 54.6	22.6 ± 56.7	0.008	22.6 ± 57.3	22.9 ± 55.4	0.928
Stent diameter (mm)	3.03 ± 0.41	3.04 ± 0.40	0.689	3.02 ± 0.38	3.11 ± 0.45	0.028
Stent length (mm)	28.8 ± 13.4	29.1 ± 14.6	0.735	28.6 ± 14.6	30.5 ± 14.6	0.192
Number of stent	1.42 ± 0.70	2.31 ± 0.99	<0.001	2.40 ± 1.00	2.03 ± 0.92	<0.001
GRACE risk score	150.9 ± 27.3	150.1 ± 26.7.	0.640	149.7 ± 26.8	151.4 ± 26.7.	0.509
>140, *n* (%)	294 (62.6)	343 (60.0)	0.394	255 (59.0)	88 (62.9)	0.422

For continuous variables, intergroup differences were evaluated with the unpaired *t*-test and data are expressed as mean ± standard deviation. For categorical variables, intergroup differences were analyzed using the χ^2^ test or, if not applicable, Fisher’s exact test and the data are expressed as count and percentage. CR, complete revascularization; IR, incomplete revascularization; LVEF, left ventricular ejection fraction; BMI, body mass index; SBP, systolic blood pressure; DBP, diastolic blood pressure; MI, myocardial infarction; PCI, percutaneous coronary intervention; CABG, coronary artery bypass graft; HF, heart failure; CVA, cerebrovascular events; CK-MB, creatine kinase myocardial band; NT-ProBNP, N-terminal pro-brain natriuretic peptide; Hs-CRP, high-sensitivity C-reactive protein; eGFR, estimated glomerular filtration rate; HDL, high-density lipoprotein; LDL, low-density lipoprotein; ACEI, angiotensin converting enzyme inhibitors; ARB, angiotensin receptor blockers; CCB, calcium channel blockers; IRA, infarct-related artery; LM, left main coronary artery; LAD, left anterior descending coronary artery; LCx, left circumflex coronary artery; RCA, right coronary artery; CAD, coronary artery disease; ACC/AHA, American College of Cardiology/American Heart Association; TIMI, thrombolysis in myocardial infarction; GP, glycoprotein; ZES, zotarolimus-eluting stent; EES, everolimus-eluting stent; BES, biolimus-eluting stent; IVUS, intravascular ultrasound; OCT, optical coherence tomography; FFR, fractional flow reserve; GRACE, Global Registry of Acute Coronary Events; ^a^ Drug-eluting stents were composed of ZES (Resolute Integrity stent; Medtronic, Inc., Minneapolis, MN), EES (Xience Prime stent, Abbott Vascular, Santa Clara, CA; or Promus Element stent, Boston Scientific, Natick, MA) and BES (BioMatrix Flex stent, Biosensors International, Morges, Switzerland; or Nobori stent, Terumo Corporation, Tokyo, Japan).

**Table 2:** Clinical outcomes.

Outcomes	Cumulative Events (%)			Unadjusted		Adjusted ^a^	
	Culprit-Only (*n* = 470)	Multivessel (*n* = 572)	Log-Rank	HR (95% CI)	*p* Value	HR (95% CI)	*p* Value
MACE	71 (16.0)	84 (15.7)	0.985	1.003 (0.731–1.376)	0.985	1.020 (0.675–1.542)	0.924
All-cause death	48 (10.8)	44 (8.2)	0.187	1.316 (0.874–1.981)	0.188	1.328 (0.774–2.278)	0.303
Cardiac death	24 (5.5)	25 (4.8)	0.606	1.159 (0.662–2.029)	0.606	1.280 (0.608–2.696)	0.516
Re-MI	14 (3.5)	22 (4.3)	0.410	0.755 (0.386–1.476)	0.412	1.178 (0.531–3.084)	0.582
Any revascularization	22 (5.3)	27 (5.3)	0.899	0.964 (0.549–1.693)	0.899	1.042 (0.496–2.188)	0.913
TVR	14 (3.4)	16 (3.1)	0.927	1.034 (0.505–2.119)	0.927	1.246 (0.490–3.167)	0.645
Non-TVR	9 (2.1)	11 (2.2)	0.968	0.982 (0.407–2.370)	0.968	1.628 (0.506–5.240)	0.414
ST (definite or probable)	4 (0.9)	9 (1.6)	0.295	0.538 (0.166–1.748)	0.303	1.367 (0.308–6.069)	0.681
**Outcomes**	**Cumulative Events (%)**			**Unadjusted**		**Adjusted ^b^**	
	**CR** **(*n* = 432)**	**IR** **(*n* = 140)**	**Log-Rank**	**HR (95% CI)**	***p* Value**	**HR (95% CI)**	***p* Value**
MACE	63 (15.5)	21 (16.6)	0.871	0.960 (0.586–1.573)	0.871	1.012 (0.577–1.776)	0.967
All-cause death	35 (8.6)	9 (7.2)	0.543	1.254 (0.603–2.610)	0.544	1.271 (0.542–2.977)	0.581
Cardiac death	18 (4.5)	7 (5.7)	0.673	0.829 (0.346–1.985)	0.674	1.429 (0.517–3.947)	0.491
Re-MI	16 (4.2)	6 (5.0)	0.719	0.842 (0.329–2.153)	0.720	1.239 (0.414–3.709)	0.702
Any revascularization	19 (5.0)	8 (6.4)	0.538	0.772 (0.338–1.764)	0.539	1.385 (0.541–3.545)	0.497
TVR	11 (2.8)	5 (4.1)	0.550	0.726 (0.252–2.089)	0.552	1.750 (0.485–6.320)	0.393
Non-TVR	8 (2.1)	3 (2.3)	0.823	0.860 (0.228–3.240)	0.823	1.524 (0.272–8.544)	0.632
ST (definite or probable)	6 (1.4)	3 (2.7)	0.533	0.646 (0.162–2.584)	0.537	1.890 (0.357–10.00)	0.454
**Outcomes**	**Cumulative Events (%)**			**Unadjusted**		**Adjusted ^c^**	
	**Culprit-Only** **(*n* = 470)**	**CR** **(*n* = 432)**	**Log-Rank**	**HR (95% CI)**	***p* Value**	**HR (95% CI)**	***p* Value**
MACE	71 (16.0)	63 (15.5)	0.956	1.010 (0.719–1.417)	0.956	1.042 (0.656–1.654)	0.863
All-cause death	48 (10.8)	35 (8.6)	0.308	1.254 (0.811–1.938)	0.309	1.223 (0.673–2.223)	0.509
Cardiac death	24 (5.5)	18 (4.5)	0.522	1.220 (0.662–2.248)	0.523	1.305 (0.569–2.993)	0.529
Re-MI	14 (3.5)	16 (4.2)	0.515	0.789 (0.385–1.616)	0.516	1.107 (0.434–2.823)	0.832
Any revascularization	22 (5.3)	19 (5.0)	0.907	1.037 (0.562–1.917)	0.907	1.096 (0.461–2.605)	0.836
TVR	14 (3.4)	11 (2.8)	0.750	1.137 (0.516–2.504)	0.751	1.906 (0.703–5.171)	0.205
Non-TVR	9 (2.1)	8 (2.1)	0.966	1.021 (0.394–2.646)	0.966	2.958 (0.683–12.81)	0.147
ST (definite or probable)	4 (0.9)	6 (1.4)	0.439	0.610 (0.172–2.161)	0.443	1.344 (0.276–6.654)	0.715
**Outcomes**	**Cumulative Events (%)**			**Unadjusted**		**Adjusted ^d^**	
	**Culprit-Only** **(*n* = 470)**	**IR** **(*n* = 140)**	**Log-Rank**	**HR (95% CI)**	***p* Value**	**HR (95% CI)**	***p* Value**
MACE	71 (16.0)	21 (16.6)	0.853	0.955 (0.587–1.554)	0.853	1.060 (0.594–1.891)	0.844
All-cause death	48 (10.8)	9 (7.2)	0.222	1.553 (0.762–3.165)	0.226	2.007 (0.882–4.569)	0.097
Cardiac death	24 (5.5)	7 (5.7)	0.993	0.996 (0.429–2.313)	0.993	1.057 (0.381–2.929)	0.916
Re-MI	14 (3.5)	6 (5.0)	0.393	0.661 (0.254–1.721)	0.396	1.807 (0.517–6.312)	0.354
Any revascularization	22 (5.3)	8 (6.4)	0.562	0.788 (0.351–1.769)	0.563	1.524 (0.542–4.280)	0.424
TVR	14 (3.4)	5 (4.1)	0.672	0.802 (0.289–2.228)	0.672	1.592 (0.405–6.264)	0.506
Non-TVR	9 (2.1)	3 (2.3)	0.850	0.882 (0.239–3.257)	0.850	1.043 (0.183–5.931)	0.962
ST (definite or probable)	4 (0.9)	3 (2.7)	0.207	0.394 (0.088–1.762)	0.223	1.446 (0.172–12.16)	0.735

HR, hazard ratio; CI, confidence interval; MACE, major adverse cardiac events; Re-MI, recurrent myocardial infarction; TVR, target vessel revascularization; ST, stent thrombosis; CR, complete revascularization; IR, incomplete revascularization; LVEF, left ventricular ejection fraction; MI, myocardial infarction; PCI, percutaneous coronary intervention; CABG, coronary artery bypass graft; CK-MB, creatine kinase myocardial band; NT-ProBNP, N-terminal pro-brain natriuretic peptide; Hs-CRP, high-sensitivity C-reactive protein; HDL, high-density lipoprotein; LDL, low-density lipoprotein; IRA, infarct-related artery; LM, left main coronary artery; LAD, left anterior descending coronary artery; LCx, left circumflex coronary artery; RCA, right coronary artery; CAD, coronary artery disease; ACC/AHA, American College of Cardiology/American Heart Association; EES, everolimus-eluting stent; IVUS, intravascular ultrasound; GRACE, Global Registry of Acute Coronary Events. ^a^ Adjusted by age, male sex, LVEF <40%, peak CK-MB, peak troponin-I, NT-ProBNP, triglyceride, cilostazole, lipid lowering agents, IRA (LM), treated vessel (LM, LAD, LCx and RCA), IVUS, time from admission to PCI, number of stent and GRACE risk score. ^b^ Adjusted by age, male sex, LVEF, hypertension, DM, previous PCI, previous CABG, peak troponin-I, NT-ProBNP, Hs-CRP, LDL-cholesterol, cilostazole, IRA (LM), treated vessel (LCx), 2-vessel disease, 3-vessel disease, ACC/AHA type B2 lesion, ZES, PCI for non-IRA, stent diameter, number of stent and GRACE risk score. ^c^ Adjusted by age, male sex, LVEF <40%, previous PCI, peak CK-MB, peak troponin-I, NT-ProBNP, HDL-cholesterol, LDL-cholesterol, cilostazole, treated vessel (LM, LAD, LCx and RCA), IVUS, time from admission to PCI and number of stent and GRACE risk score. ^d^ Adjusted by age, male sex, LVEF, DM, peak troponin-I, NT-ProBNP, blood glucose, lipid lowering agent, IRA (LM), treated vessel (LM, LAD, LCx and RCA), 2-vessel disease, 3-vessel disease, ACC/AHA type B2 lesion, EES, IVUS, stent diameter, number of stent and GRACE risk score.

**Table 3:** Independent predictors for MACE.

Variables	Unadjusted		Adjusted	
	HR (95% CI)	*p*	HR (95% CI)	*p*
C-PCI vs. M-PCI	1.003 (0.731–1.376)	0.985	1.090 (0.706–1.684)	0.696
CR vs. IR	0.960 (0.586–1.573)	0.871	1.021 (0.552–1.888)	0.947
C-PCI vs. CR	1.010 (0.719–1.417)	0.956	1.192 (0.741–1.920)	0.469
C-PCI vs. IR	0.955 (0.587–1.554)	0.853	1.143 (0.592–2.206)	0.691
Age, ≥65 years	1.438 (1.019–2.030)	0.039	1.422 (0.951–2.010)	0.083
Male	1.317 (0.954–1.820)	0.095	1.282 (0.894–1.838)	0.177
LVEF, <40%	1.993 (1.438–2.763)	<0.001	1.482 (1.026–2.235)	0.029
Killip class III	1.691 (1.180–2.423)	0.004	1.349 (0.836–2.177)	0.220
Hypertension	1.169 (0.792–1.724)	0.432	1.146 (0.756–1.737)	0.520
Diabetes mellitus	1.471 (1.060–2.041)	0.021	1.387 (0.948–2.028)	0.092
Previous PCI	1.105 (0.704–1.735)	0.665	1.080 (0.667–1.748)	0.756
Previous CABG	1.409 (0.522–3.803)	0.499	1.451 (0.520–4.050)	0.477
Peak CK-MB	1.001 (1.000–1.002)	0.186	1.001 (1.000–1.002)	0.192
Peak troponin-I	1.001 (1.001–1.002)	<0.001	1.002 (1.001–1.002)	0.002
NT-ProBNP	1.000 (0.999–1.001)	0.001	1.001 (1.000–1.002)	0.026
Hs-CRP	0.998 (0.993–1.004)	0.550	0.997 (0.990–1.003)	0.325
Blood glucose	1.000 (0.999–1.002)	0.790	0.999 (0.997–1.001)	0.214
Total cholesterol	0.999 (0.995–1.002)	0.425	0.999 (0.995–1.004)	0.833
Triglyceride	0.999 (0.997–1.001)	0.445	0.999 (0.976–1.002)	0.527
HDL-cholesterol	0.979 (0.964–0.995)	0.008	0.980 (0.970–1.001)	0.051
LDL-cholesterol	1.002 (0.998–1.005)	0.397	1.002 (0.998–1.007)	0.353
Ticagrelor	1.228 (0.574–2.628)	0.596	1.436 (0.650–3.174)	0.371
Prasugrel	1.422 (0.527–3.839)	0.488	1.144 (0.398–3.288)	0.802
Cilostazole	1.238 (0.862–1.780)	0.248	1.293 (0.874–1.911)	0.198
ACEI	1.098 (0.796–1.514)	0.569	1.014 (0.660–1.559)	0.949
ARB	1.067 (0.766–1.484)	0.702	1.066 (0.691–1.645)	0.722
Beta-blocker	1.166 (0.783–1.737)	0.449	1.132 (0.731–1.753)	0.578
Lipid lowering agent	1.057 (0.722–1.546)	0.775	1.168 (0.768–1.776)	0.467
LM-IRA	1.451 (0.786–2.679)	0.234	1.004 (0.304–3.312)	0.995
LM-treated vessel	1.398 (0.821–2.381)	0.218	1.444 (0.522–3.993)	0.179
LAD-treated vessel	1.036 (0.752–1.429)	0.827	1.069 (0.743–1.538)	0.719
LCx-treated vessel	1.076 (0.784–1.478)	0.649	1.133 (0.791–1.622)	0.497
RCA-treated vessel	1.153 (0.842–1.580)	0.375	1.184 (0.812–1.725)	0.369
ACC/AHA type B2/C lesion	1.263 (0.834–1.913)	0.270	1.105 (0.709–1.721)	0.680
IVUS	1.041 (0.695–1.559)	0.844	1.038 (0.681–1.582)	0.862
Time from admission to PCI	1.001 (0.998–1.003)	0.193	1.091 (1.000–1.271)	0.080
Stent diameter <3.0 mm	0.900 (0.647–1.253)	0.533	1.171 (0.820–1.671)	0.385
Stent length ≥30 mm	1.417 (1.026–1.957)	0.034	1.379 (0.968–1.966)	0.075
GRACE risk score	1.071 (1.012–1.031)	0.037	1.001 (0.993–1.010)	0.778

MACE, major adverse cardiac events; HR, hazard ratio; CI, confidence interval; C-PCI, culprit-only PCI, M-PCI, multivessel PCI; CR, complete revascularization; IR, incomplete revascularization; PCI, percutaneous coronary intervention; LVEF, left ventricular ejection fraction; CABG, coronary artery bypass graft; CK-MB, creatine kinase myocardial band; NT-ProBNP, N-terminal pro-brain natriuretic peptide; Hs-CRP, high-sensitivity C-reactive protein; HDL, high-density lipoprotein; LDL, low-density lipoprotein; ACEI, angiotensin converting enzyme inhibitor; ARB, angiotensin receptor blocker; LM, left main coronary artery; IRA, infarct-related artery; LAD, left anterior descending coronary artery; LCx, left circumflex coronary artery; RCA, right coronary artery; ACC/AHA, American College of Cardiology/American Heart Association; IVUS, intravascular ultrasound; GRACE, Global Registry of Acute Coronary Events.

## Data Availability

Data is contained within the article or supplementary material.

## Supplementary Materials

The following are available online at https://www.mdpi.com/article/10.3390/jcm10204629/s1, Table S1: Baseline clinical, laboratory, angiographic and procedural characteristics, Figure S1: Kaplan-Meier analyses for the MACE (A), all-cause death (B), cardiac death (C), recurrent MI (D), any repeat revascularization (E), TVR (F), non-TVR (G) and stent thrombosis (H) between the C-PCI group and the CR group and the C-PCI group and the IR group at 2 years. MACE, major adverse cardiac events; MI, myocardial infarction; TVR, target vessel revascularization; C-PCI, culprit-only PCI; M-PCI, multivessel PCI; CR, complete revascularization; IR, incomplete revascularization.
