# Supplementary material for: Outcomes of Different Reperfusion Strategies of Multivessel Disease Undergoing Newer-Generation Drug-Eluting Stent Implantation in Patients with Non-ST-Elevation Myocardial Infarction and Chronic Kidney Disease
Source: J Clin Med. 2021 Oct 9;10(20):4629. doi: 10.3390/jcm10204629 (PMC8539165; doi:10.3390/jcm10204629)
Supplement: Supplementary file 1 [file jcm-10-04629-s001.zip › supplementary Figure S1.pptx]

## Slide 1
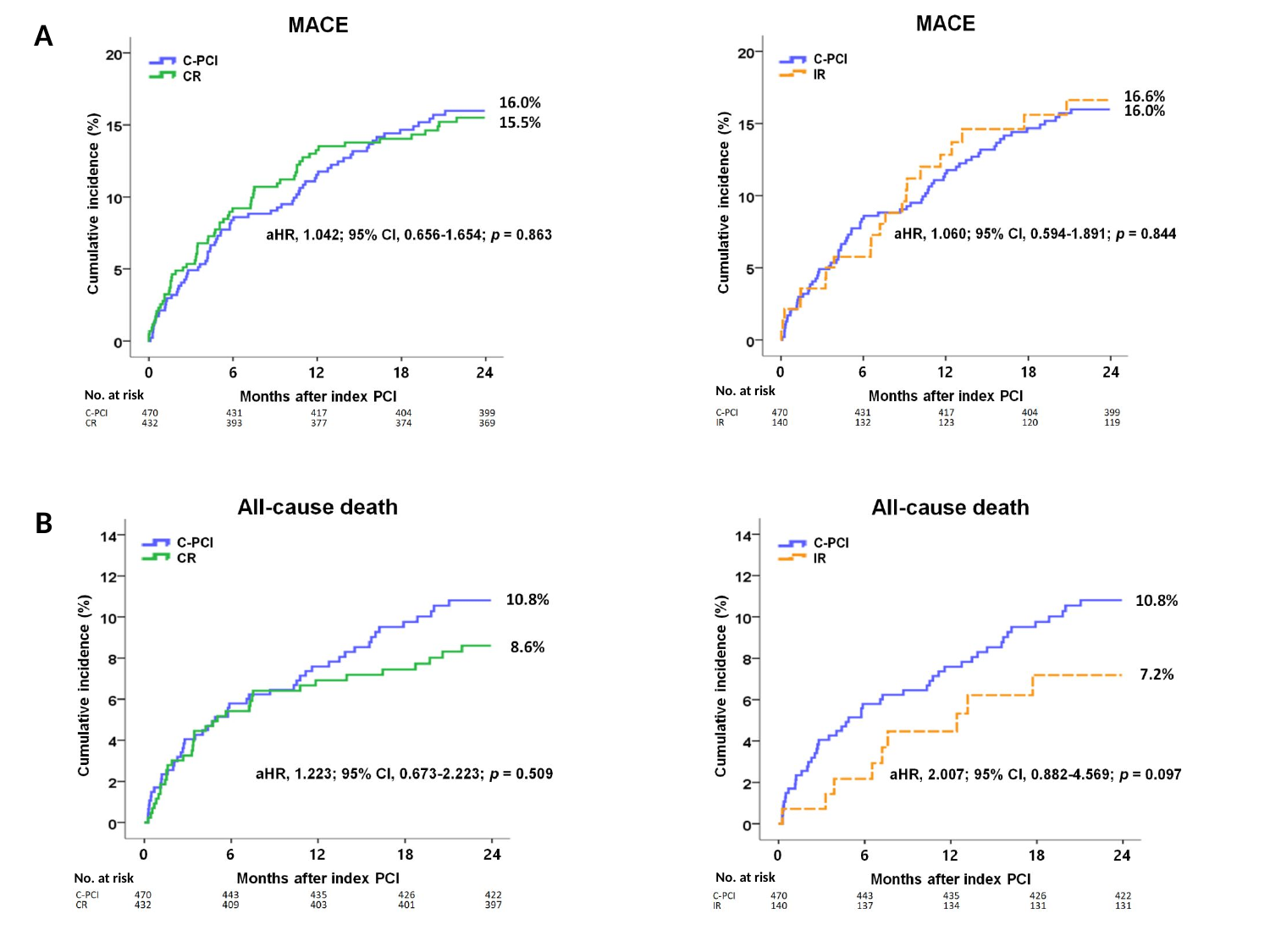

A
No. at risk
No. at risk
B
No. at risk
No. at risk

## Slide 2
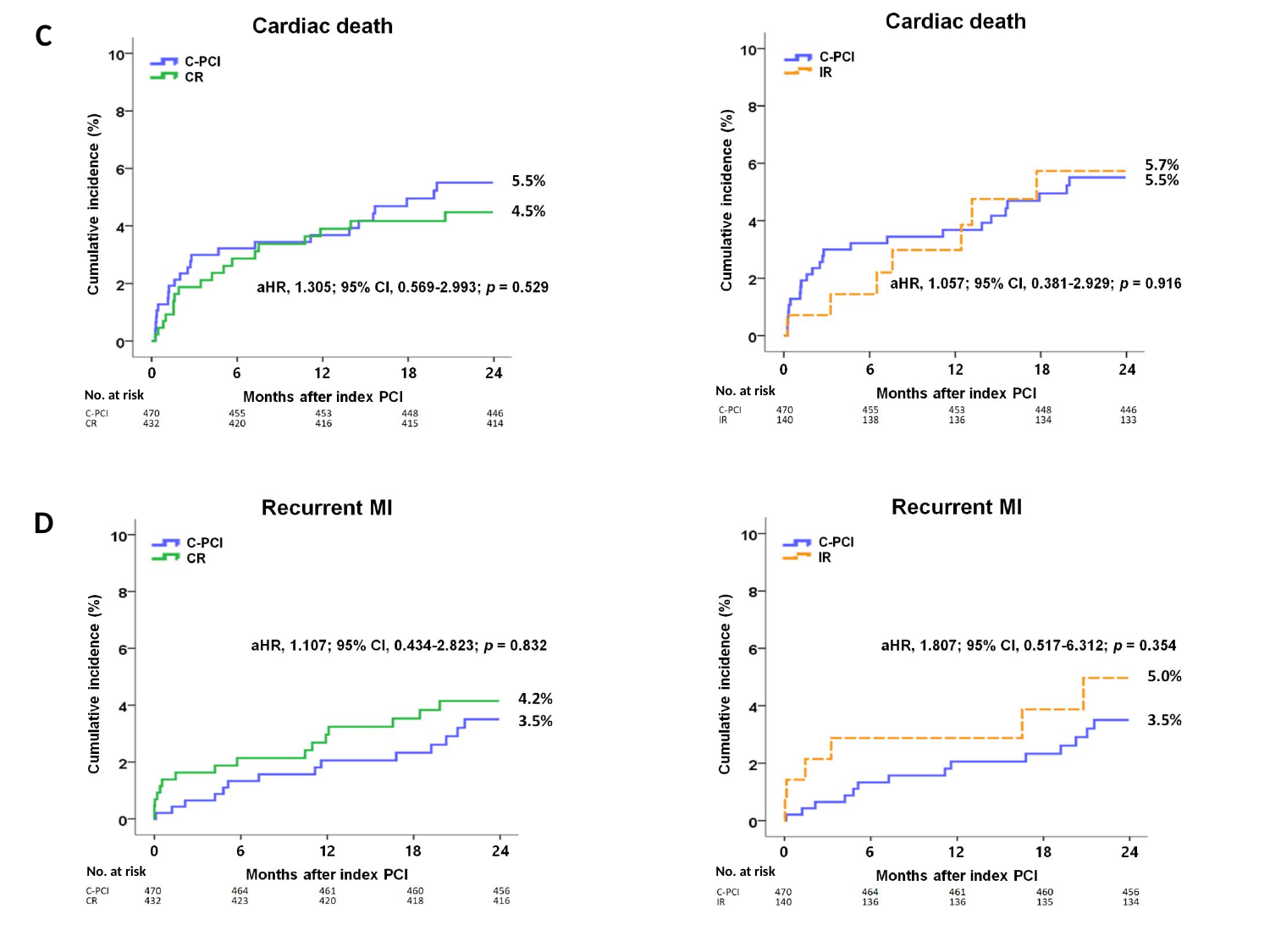

C
No. at risk
No. at risk
D
No. at risk
No. at risk

## Slide 3
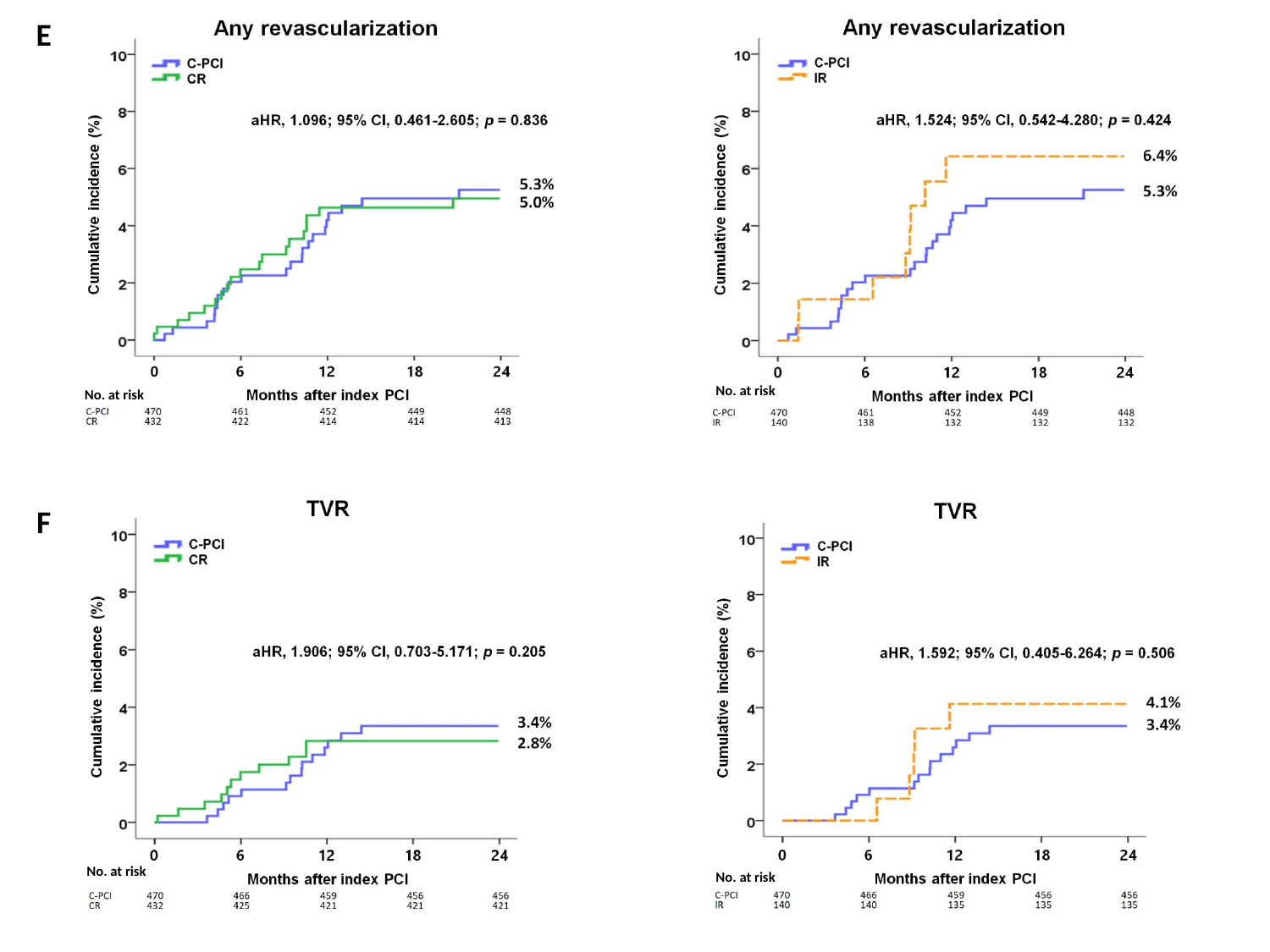

E
No. at risk
No. at risk
F
No. at risk
No. at risk

## Slide 4
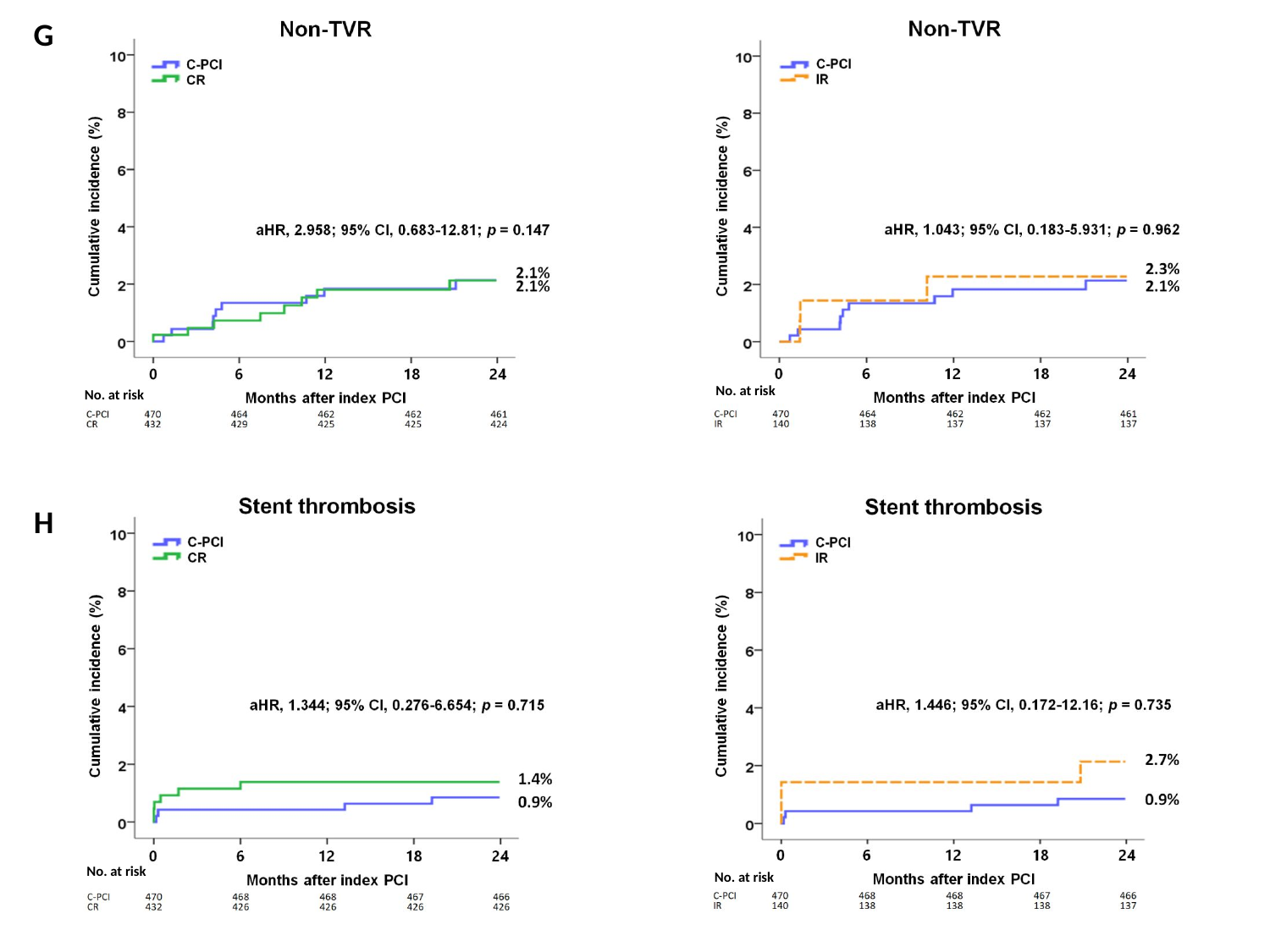

G
No. at risk
No. at risk
H
No. at risk
No. at risk
