# Supplementary material for: Outcomes of Different Reperfusion Strategies of Multivessel Disease Undergoing Newer-Generation Drug-Eluting Stent Implantation in Patients with Non-ST-Elevation Myocardial Infarction and Chronic Kidney Disease
Source: J Clin Med. 2021 Oct 9;10(20):4629. doi: 10.3390/jcm10204629 (PMC8539165; doi:10.3390/jcm10204629)
Supplement: Supplementary file 1 [file jcm-10-04629-s001.zip › supplementary Table S1.docx]

**Table S1.** Baseline clinical, laboratory, angiographic, and procedural characteristics

| Variables | C-PCI  (*n* = 470) | CR  (*n* = 432) | *p* value | C-PCI  (*n* = 470) | IR  (*n* = 140) | *p* value |
| --- | --- | --- | --- | --- | --- | --- |
| Age (years) | 71.7 ± 9.7 | 71.3 ± 9.2 | 0.495 | 71.7 ± 9.7 | 71.2 ± 9.0 | 0.521 |
| ≥65 years, *n* (%) | 364 (77.4) | 328 (75.9) | 0.589 | 364 (77.4) | 106 (75.7) | 0.669 |
| Male, *n* (%) | 278 (59.1) | 217 (50.2) | 0.007 | 278 (59.1) | 81 (57.9) | 0.785 |
| LVEF (%) | 48.3 ± 12.6 | 49.9 ± 12.7 | 0.054 | 48.3 ± 12.6 | 46.1 ± 12.5 | 0.037 |
| <40%, *n* (%) | 116 (24.7) | 93 (21.5) | 0.262 | 116 (24.7) | 38 (27.1) | 0.556 |
| BMI (kg/m^2^) | 23.6 ± 3.3 | 23.9 ± 3.3 | 0.133 | 23.6 ± 3.3 | 23.54 ±3.3 | 0.456 |
| SBP (mmHg) | 133.9 ± 30.1 | 134.5 ± 29.3 | 0.762 | 133.9 ± 30.1 | 135.3 ± 31.0 | 0.624 |
| DBP (mmHg) | 78.3 ± 16.6 | 78.1 ± 15.4 | 0.844 | 78.3 ± 16.6 | 78.3 ± 15.6 | 0.974 |
| Killip class III, *n* (%) | 83 (17.7) | 75 (17.4) | 0.930 | 83 (17.7) | 26 (18.6) | 0.802 |
| Hypertension, *n* (%) | 363 (77.2) | 323 (74.8) | 0.386 | 363 (77.2) | 116 (82.9) | 0.155 |
| Diabetes mellitus, *n* (%) | 247 (52.6) | 234 (54.2) | 0.628 | 247 (52.6) | 91 (65.0) | 0.009 |
| Dyslipidemia, *n* (%) | 294 (62.6) | 294 (68.1) | 0.083 | 294 (62.6) | 84 (60.0) | 0.585 |
| Previous MI, *n* (%) | 37 (7.9) | 33 (7.6) | 0.902 | 37 (7.9) | 15 (10.7) | 0.302 |
| Previous PCI, *n* (%) | 71 (15.1) | 42 (9.7) | 0.016 | 71 (15.1) | 26 (18.6) | 0.325 |
| Previous CABG, *n* (%) | 11 (2.3) | 3 (0.7) | 0.059 | 11 (2.3) | 5 (3.6) | 0.382 |
| Previous HF, *n* (%) | 22 (4.7) | 18 (4.2) | 0.748 | 22 (4.7) | 7 (5.0) | 0.824 |
| Previous CVA, *n* (%) | 72 (15.3) | 54 (12.5) | 0.249 | 72 (15.3) | 19 (13.6) | 0.686 |
| Current smokers, *n* (%) | 98 (20.9) | 80 (18.5) | 0.403 | 98 (20.9) | 23 (16.4) | 0.278 |
| Peak CK-MB (mg/dL) | 61.1 ± 96.8 | 47.6 ± 75.9 | 0.035 | 61.1 ± 96.8 | 52.5 ± 94.5 | 0.368 |
| Peak troponin-I (ng/mL) | 35.6 ± 91.5 | 24.4 ± 60.0 | 0.084 | 35.6 ± 91.5 | 34.3 ± 88.7 | 0.834 |
| NT-ProBNP (pg/mL) | 7027.3 ± 9781.9 | 5825.1 ± 8862.6 | 0.069 | 7027.3 ± 9781.9 | 7015.4 ± 8725.4 | 0.765 |
| Hs-CRP (mg/dL) | 9.4 ± 32.9 | 10.8 ± 45.8 | 0.600 | 9.4 ± 32.9 | 5.5 ± 17.4 | 0.067 |
| Serum creatinine (mg/L) | 2.41 ± 2.45 | 2.32 ± 2.64 | 0.621 | 2.41 ± 2.45 | 2.45 ± 2.58 | 0.883 |
| eGFR, mL/min/1.73m^2^ | 39.6 ± 16.7 | 40.8 ± 16.5 | 0.305 | 39.6 ± 16.7 | 38.3 ± 16.8 | 0.390 |
| Blood glucose (mg/dL) | 189.4 ± 100.3 | 193.6 ± 108.1 | 0.543 | 189.4 ± 100.3 | 213.6 ± 114.7 | 0.025 |
| Total cholesterol (mg/dL) | 169.7 ± 56.8 | 174.1 ± 45.7 | 0.193 | 169.7 ± 56.8 | 172.1 ± 48.1 | 0.612 |
| Triglyceride (mg/L) | 118.2 ± 71.0 | 130.9 ± 116.3 | 0.052 | 118.2 ± 71.0 | 121.8 ± 66.0 | 0.580 |
| HDL cholesterol (mg/L) | 42.5 ± 22.2 | 40.2 ± 10.5 | 0.038 | 42.5 ± 22.2 | 42.0 ± 12.0 | 0.738 |
| LDL cholesterol (mg/L) | 103.8 ± 41.9 | 109.1 ± 35.4 | 0.040 | 103.8 ± 41.9 | 101.5 ± 38.0 | 0.542 |
| Discharge medications |  |  |  |  |  |  |
| Aspirin, *n* (%) | 451 (96.0) | 418 (96.8) | 0.522 | 451 (96.0) | 136 (97.1) | 0.518 |
| Clopidogrel, *n* (%) | 435 (92.6) | 405 (93.8) | 0.655 | 435 (92.6) | 125 (89.3) | 0.214 |
| Ticagrelor, *n* (%) | 23 (4.9) | 19 (4.4) | 0.754 | 23 (4.9) | 12 (8.6) | 0.144 |
| Prasugrel, *n* (%) | 12 (2.6) | 8 (1.9) | 0.506 | 12 (2.6) | 3 (2.1) | 0.783 |
| Cilostazole, *n* (%) | 77 (16.4) | 121 (28.0) | <0.001 | 77 (16.4) | 17 (12.1) | 0.286 |
| Beta-blocker, *n* (%) | 368 (78.3) | 339 (78.5) | 0.949 | 368 (78.3) | 110 (78.6) | 0.945 |
| ACEI, *n* (%) | 202 (43.0) | 180 (41.7) | 0.690 | 202 (43.0) | 53 (37.9) | 0.329 |
| ARB, *n* (%) | 167 (35.5) | 158 (36.6) | 0.745 | 167 (35.5) | 56 (40.0) | 0.368 |
| CCB, *n* (%) | 81 (17.2) | 64 (14.8) | 0.364 | 81 (17.2) | 24 (17.1) | 0.980 |
| Lipid lowering agents, *n* (%) | 360 (76.6) | 348 (80.6) | 0.148 | 360 (76.6) | 122 (87.1) | 0.007 |
| Angiographic & procedural characteristics | | | | | | |
| IRA |  |  |  |  |  |  |
| Left main, *n* (%) | 17 (3.6) | 22 (5.1) | 0.326 | 17 (3.6) | 14 (10.0) | 0.007 |
| LAD, *n* (%) | 190 (40.4) | 151 (35.0) | 0.189 | 190 (40.4) | 51 (36.4) | 0.421 |
| LCx, *n* (%) | 105 (22.3) | 114 (26.4) | 0.157 | 105 (22.3) | 29 (20.7) | 0.683 |
| RCA, *n* (%) | 158 (33.6) | 145 (33.6) | 0.987 | 158 (33.6) | 46 (32.9) | 0.867 |
| Treated vessel |  |  |  |  |  |  |
| Left main, *n* (%) | 21 (4.5) | 38 (8.8) | 0.010 | 21 (4.5) | 18 (12.9) | 0.001 |
| LAD, *n* (%) | 217 (46.2) | 321 (74.3) | <0.001 | 217 (46.2) | 99 (70.7) | <0.001 |
| LCx, *n* (%) | 131 (27.9) | 284 (65.7) | <0.001 | 131 (27.9) | 68 (48.6) | <0.001 |
| RCA, *n* (%) | 180 (38.3) | 247 (57.2) | <0.001 | 180 (38.3) | 70 (50.0) | 0.013 |
| Extent of CAD |  |  |  |  |  |  |
| 2-vessel disease, *n* (%) | 229 (48.7) | 227 (52.5) | 0.258 | 229 (48.7) | 43 (30.7) | <0.001 |
| ≥ 3-vessel disease, *n* (%) | 241 (51.3) | 205 (47.5) | 0.258 | 241 (51.3) | 97 (69.3) | <0.001 |
| ACC/AHA lesion type |  |  |  |  |  |  |
| Type B1, *n* (%) | 62 (13.2) | 54 (12.5) | 0.766 | 62 (13.2) | 24 (17.1) | 0.238 |
| Type B2, *n* (%) | 154 (32.8) | 154 (35.6) | 0.362 | 154 (32.8) | 26 (18.6) | 0.001 |
| Type C, *n* (%) | 224 (47.7) | 196 (45.4) | 0.491 | 224 (47.7) | 76 (54.3) | 0.169 |
| Pre-PCI TIMI flow grade 0/1, *n* (%) | 185 (39.4) | 179 (41.4) | 0.526 | 185 (39.4) | 49 (35.0) | 0.352 |
| In-hospital GP IIb/IIIa, *n* (%) | 28 (6.0) | 17 (3.9) | 0.172 | 28 (6.0) | 8 (5.7) | 0.915 |
| Drug-eluting stents^a^ |  |  |  |  |  |  |
| ZES, *n* (%) | 168 (35.7) | 164 (38.0) | 0.534 | 168 (35.7) | 39 (27.9) | 0.085 |
| EES, *n* (%) | 248 (52.8) | 233 (53.9) | 0.725 | 248 (52.8) | 88 (62.9) | 0.035 |
| BES, *n* (%) | 54 (11.5) | 47 (10.9) | 0.833 | 54 (11.5) | 19 (13.6) | 0.505 |
| Others, *n* (%) | 6 (1.3) | 6 (1.4) | 0.883 | 6 (1.3) | 1 (0.7) | 0.583 |
| IVUS, *n* (%) | 68 (14.5) | 99 (22.9) | 0.001 | 68 (14.5) | 39 (27.9) | <0.001 |
| OCT, *n* (%) | 1 (0.2) | 1 (0.2) | 0.952 | 1 (0.2) | 1 (0.7) | 0.407 |
| FFR, *n* (%) | 3 (0.6) | 1 (0.2) | 0.626 | 3 (0.6) | 1 (0.7) | 0.922 |
| Completeness of multivessel PCI |  |  |  |  |  |  |
| CR, *n* (%) | - | 432 (100.0) | - | - | - | - |
| IR, *n* (%) |  | - | - |  | 140 (100.0) | - |
| PCI for non-IRA | - |  |  | - |  |  |
| During index PCI, *n* (%) | - | 315 (72.9) | - | - | 87 (62.1) |  |
| Staged PCI before discharge, *n* (%) | - | 117 (27.1) | - | - | 53 (37.9) |  |
| Time from admission to PCI (hours) | 18.1 ± 54.6 | 22.6 ± 57.3 | 0.008 | 18.1 ± 54.6 | 22.9 ± 55.4 | 0.007 |
| Stent diameter (mm) | 3.03 ± 0.41 | 3.02 ± 0.38 | 0.622 | 3.03 ± 0.41 | 3.11 ± 0.45 | 0.060 |
| Stent length (mm) | 28.8 ± 13.4 | 28.6 ± 14.6 | 0.864 | 28.8 ± 13.4 | 30.5 ± 14.6 | 0.220 |
| Number of stent | 1.42 ± 0.70 | 2.40 ± 1.00 | <0.001 | 1.42 ± 0.70 | 2.03 ± 0.92 | <0.001 |
| GRACE risk score | 150.9 ± 27.3 | 149.7 ± 26.8 | 0.501 | 150.9 ± 27.3 | 151.4 ± 26.7. | 0.847 |
| > 140, *n* (%) | 294 (62.6) | 255 (59.0) | 0.278 | 294 (62.6) | 88 (62.9) | 0.885 |

For continuous variables, intergroup differences were evaluated with the unpaired t-test and data are expressed as mean ± standard deviation. For categorical variables, intergroup differences were analyzed using the χ^2^ test or, if not applicable, Fisher’s exact test and the data are expressed as count and percentage. CR, complete revascularization; IR, incomplete revascularization; LVEF, left ventricular ejection fraction; BMI, body mass index; SBP, systolic blood pressure; DBP, diastolic blood pressure; MI, myocardial infarction; PCI, percutaneous coronary intervention; CABG, coronary artery bypass graft; HF, heart failure; CVA, cerebrovascular events; CK-MB, creatine kinase myocardial band; NT-ProBNP, N-terminal pro-brain natriuretic peptide; Hs-CRP, high-sensitivity C-reactive protein; eGFR, estimated glomerular filtration rate; HDL, high-density lipoprotein; LDL, low-density lipoprotein; ACEI, angiotensin converting enzyme inhibitors; ARB, angiotensin receptor blockers; CCB, calcium channel blockers; IRA, infarct-related artery; LM, left main coronary artery; LAD, left anterior descending coronary artery; LCx, left circumflex coronary artery; RCA, right coronary artery; CAD, coronary artery disease; ACC/AHA, American College of Cardiology/American Heart Association; TIMI, thrombolysis in myocardial infarction; GP, glycoprotein; ZES, zotarolimus-eluting stent; EES, everolimus-eluting stent; BES, biolimus-eluting stent; IVUS, intravascular ultrasound; OCT, optical coherence tomography; FFR, fractional flow reserve; CR, complete revascularization; IR, incomplete revascularization; ^a^Drug-eluting stents were composed of ZES (Resolute Integrity stent; Medtronic, Inc., Minneapolis, MN), EES (Xience Prime stent, Abbott Vascular, Santa Clara, CA; or Promus Element stent, Boston Scientific, Natick, MA), and BES (BioMatrix Flex stent, Biosensors International, Morges, Switzerland; or Nobori stent, Terumo Corporation, Tokyo, Japan).
